# Study of microbiome changes in patients with ulcerative colitis in the Central European part of Russia

**DOI:** 10.1016/j.heliyon.2021.e06432

**Published:** 2021-03-10

**Authors:** M.V. Gryaznova, S.A. Solodskikh, A.V. Panevina, M.Y. Syromyatnikov, Yu.D. Dvoretskaya, T.N. Sviridova, E.S. Popov, V.N. Popov

**Affiliations:** a Department of Genetics, Cytology and Bioengineering, Voronezh State University, 394018 Voronezh, Russia; b Laboratory of Metagenomics and Food Biotechnology, Voronezh State University of Engineering Technologies, 394036 Voronezh, Russia; c Department of Hospital Therapy and Endocrinology, Voronezh State Medical University Named After N.N. Burdenko, 394036 Voronezh, Russia; d Family Medicine Center "Olympus of Health", 394036 Voronezh, Russia

**Keywords:** Intestinal microbiota, Ulcerative colitis, Inflammatory bowel disease, Sequencing, Russian population

## Abstract

Ulcerative colitis (UC) is an inflammatory disease that affects the colon and rectum. Recently, evidence has emerged about the influence of microbiota on the development of this disease. However, studies on the role of intestinal microbiota in the pathogenesis of UC have been incomplete. In addition, there are no comprehensive studies of the causes of ulcerative colitis and data on the microbiological composition of the intestines of patients with ulcerative colitis in Russia. We carried out a study of the microbiological composition of the intestines of patients with ulcerative colitis and healthy individuals. We found significant changes in the bacteria genera and species in patients with UC compared with the control group using sequencing on the IonTorrent PGM system and subsequent data analysis. In our study we observed a significant increase of the genus *Haemophilus, Olsenella, Prevotella, Cedecea, Peptostreptococcus, Faecalibacterium, Lachnospira, Negativibacillus, Butyrivibrio*, and the species *Bacteroides coprocola, Phascolarctobacterium succinatutens, Dialister succinatiphilus, Sutterella wadsworthensis, Faecalibacterium prausnitzii* in patients with ulcerative colitis. In addition, in patients with ulcerative colitis there was a significant decrease in the genus *Fusicatenibacter, Butyricimonas, Lactococcus, Eisenbergiella, Coprobacter, Cutibacterium, Falsochrobactrum, Brevundimonas, Yersinia, Leuconostoc* and in the species *Fusicatenibacter saccharivorans*. We found confirmation of our data with literary sources and studies of UC. In addition, we discovered a few taxa such as *Negativibacillus* spp. and *Falsochrobactrum* spp. that have not been previously found in human stool samples. Our data confirm that more research is needed to understand the role of microbiome changes in the development of UC in different people populations.

## 1 Introduction

Ulcerative colitis (UC) is a chronic inflammatory bowel disease (IBD) of the colon that continuously causes superficial inflammation of the mucous membrane, extending from the rectum to the more proximal part of the colon [1]. The characteristic symptoms of UC include bloody diarrhea with urgency of the rectum and tenesmus. The following are risk factors for UC: age and gender, race and ethnicity (Jewish population has higher risk of developing IBD than non-Jewish population), genetics (approximately 8–14% of ulcerative colitis patients have a family history of IBD), smoking, diet ("Western" style diet is associated with an increased risk of developing IBD), appendectomy [2]. Recently, evidence has emerged about the influence of microbiota on the development of this disease. The transcriptional profile of the mucosa has been shown to interact with the microbiota of the colon. Bacterial functions, such as the production of butyrate can affect gene expression of the mucosa. It was also shown that patients with UC had a lower percentage of potentially protective bacteria species than their healthy twins [3]. In patients with UC, the intestinal microbial population as well as the functional diversity and stability of intestinal bacteria are impaired, leading to a decrease in the number of specific Firmicutes bacteria and an increase in the number of Bacteroidetes bacteria and facultative anaerobes [4]. Several studies have shown a link between UC and *A. muciniphila*. *A. muciniphila* has been reduced in UC patients [5]. Two genus of bacteria – *Desulfovibrio* and *Clostridium* has been closely related to UC [6]. In patients with UC, there is a decrease in the intestinal population of representatives of Firmicutes and Bacteroidetes phyla, as well as an increase in the population of *Lactobacillus* [7]. The richness, uniformity, and biological diversity of the intestinal microbiome were markedly reduced in children with UC compared to healthy controls [8]. Toxins of *Clostridium difficile* can play an active role in the pathogenesis of ulcerative colitis [9]. Some microbial pathogens may be associated with intestinal inflammation, and thus patients with UC may harbor *Mycobacterium avium paratuberculosis*, adhesive-invasive *Escherichia coli*, *Helicobacter* sp*.*, *Salmonella* sp*.*, *Yersinia* sp., *Fusobacterium* sp., *Listeria* sp. and norovirus species [10].

It was found that ulcerative colitis changes the community of viruses. UC is characterized by substantial changes in the mucous virobiota with functional distortion [11]. There is evidence that fecal microbiota transplantation can positively effect on patients with ulcerative colitis [12, 13, 14].

Currently, the incidence and prevalence of UC has not only clearly increased in Europe and North America [15], but has also grown rapidly in Asian countries [16, 17]. There are no comprehensive studies of the causes of ulcerative colitis in Russia. There are no data on the microbiological composition of the intestines of patients with ulcerative colitis in Russia. The aim of this work was to study the microbiological composition of the intestines of patients with ulcerative colitis in the Central European part of Russia.

## 2 Materials and methods

### 2.1 Samples

Fecal samples were collected at the “Olympus of Health” Clinic (Voronezh, Russia). Table 1 provides detailed information on patients with UC.

**Table 1:** Information on patients with UC.

Sample ID	Gender	Date of Birth	Duration of UC (years)	Diagnostic methods
2.1	Male	1.3.1971	3	Colonoscopy with biopsy; morphological examination of biopsies of the colon mucosa; fecal calprotectin study; coprogram; general clinical blood test; blood biochemistry.
2.2	Female	29.10.1988	5	
2.3	Male	15.9.1981	13	
2.4	Female	16.2.1964	4	
2.5	Female	12.7.1949	26	
2.6	Male	16.9.1986	1	
2.7	Male	10.6.1999	2	
2.8	Male	7.8.1994	4	
2.9	Male	21.8.1964	7	
2.10	Female	03.07.1983	5	

One fecal sample (450 mg–1000 mg) was taken from each patient for analysis.

### 2.2 DNA isolation

DNA was isolated from the collected fecal samples using a ZymoBIOMICS DNA Microprep Kit (Zymo research, USA) as recommended by the manufacturer (https://files.zymoresearch.com/protocols/_d4301_d4305_zymobiomics_dna_microprep_kit.pdf).

### 2.3 PCR

Bacterial DNA was amplified with the universal direct 785F forward primer (GGATTAGATACCCTGGTA) and reverse 1100R primer (GGGTTGCGCTCGTTG) [18]. PCR was performed using a 5X ScreenMix-HS Master Mix (Evrogen, Russia) in the following regime: 94°С for 4 min followed by 37 cycles of 94°С for 30 s, 53°С for 30 s, and 72°С for 30 s with the final elongation at 72°С for 5 min.

### 2.4 High-throughput sequencing

PCR products were purified with AMPure XP magnetic beads (Beckman Coulter, USA) and used for constructing sequencing libraries using Ion AmpliSeq Library Kit 2.0 (Thermo Fisher Scientific, USA) as recommended by the manufacturer. Barcoding was performed using Ion Xpress barcode adapters (Thermo Fisher Scientific, USA). Library DNA concentration was determined by qPCR using Library Quantification Kit Ion Torrent Platforms (Kapa Biosystems, USA).

Sequencing was performed on the IonTorrent PGM system using Ion PGM Hi-Q View Sequencing Kit, Ion OneTouch 2 System, and Ion PGM Hi-Q View OT2 Kit (Thermo Fisher Scientific, USA).

### 2.5 Data analysis

Sequencing results were obtained as binary alignment map (BAM) files that were converted into the FASTQ format using the SAMtools v.1.2 software [19]. Demultiplexing was done with the fastq-multx application of the ea-utils v.1.3 program package [20]. The reads were filtered according to the reading quality based on the expected number of errors using the maximum expected error cutoff 1.0 [21]. The samples were pooled and unique sequences were identified before searching for the operational taxonomic units (OTUs). We searched for the OTUs using the UNOISE2 algorithm that reduces the noise through error correction [22]. We combined all reads for all samples, for generating OTUs and making an OTU table. The most important reason for pooling is that it enhances the abundance signal for correct sequences. If samples are pooled, then a sequence that appears as a singleton in one sample may also appear in another sample and will therefore be retained and included in the OTU table. If singletons are discarded after pooling (as usually recommended in order to reduce spurious OTUs), then more low-abundance species will be retained compared with discarding singletons for each sample separately.

Filtration of reads, identification of unique sequences, and clusterization in order to search for the OTUs were performed using either USEARCH v.10.0.240 or VSEARCH v.2.8.2 software. Microbial species in the samples were identified using the SILVA database v.123 (https://www.arb-silva.de).

In order to compare relative abundances between different experimental groups, we used generalized linear modelling (GLM) method [23] implemented in DeSEQ2 R package [24]. Briefly, final estimate of logarithmic fold changes for each OTU, performed by DeSEQ2, is based on gene-wise dispersion estimates comparison. The starting point of a DESeq2 analysis is a count matrix K with one row for each taxa *i* and one column for each sample *j*. The matrix entries *K*_*ij*_ indicate the size of the OTU. DeSEQ2 algorithm models read counts *K*_*ij*_ as following a negative binomial distribution with mean *μ*_*ij*_ and dispersion *α*_*i*_. The mean is taken as a quantity *q*_*ij*_, proportional to the concentration of 16s rRNA DNA fragments from the microorganism in the sample, scaled by a normalization factor:

*s*_*ij*_, i.e., μ_*ij*_ = *s*_*ij*_*q*_*ij*_

DeSEQ2 uses GLMs with a logarithmic link,

$${\log\;}_{2}{\text{q}}_{\text{ij}}=\underset{\text{r}}{\sum }{\text{x}}_{\text{jr}}{\text{β}}_{\text{ir}}$$

with design matrix elements *x*_*jr*_ and coefficients *β*_*ir*_. In our case, a comparison between two groups (e.g. control vs UC or control vs IBD) was performed, which produces the design matrix, where elements indicate whether a sample *j* belongs to experimental group (UC or IBD), or not. P values for each OTU are obtained using Wald test.

### 2.6 Ethics and patient consent guidelines

All patients, before undergoing examination in the clinic, give their written consent to the use of their anonymized personal data for research purposes.

## 3 Results

**Figure 1:**
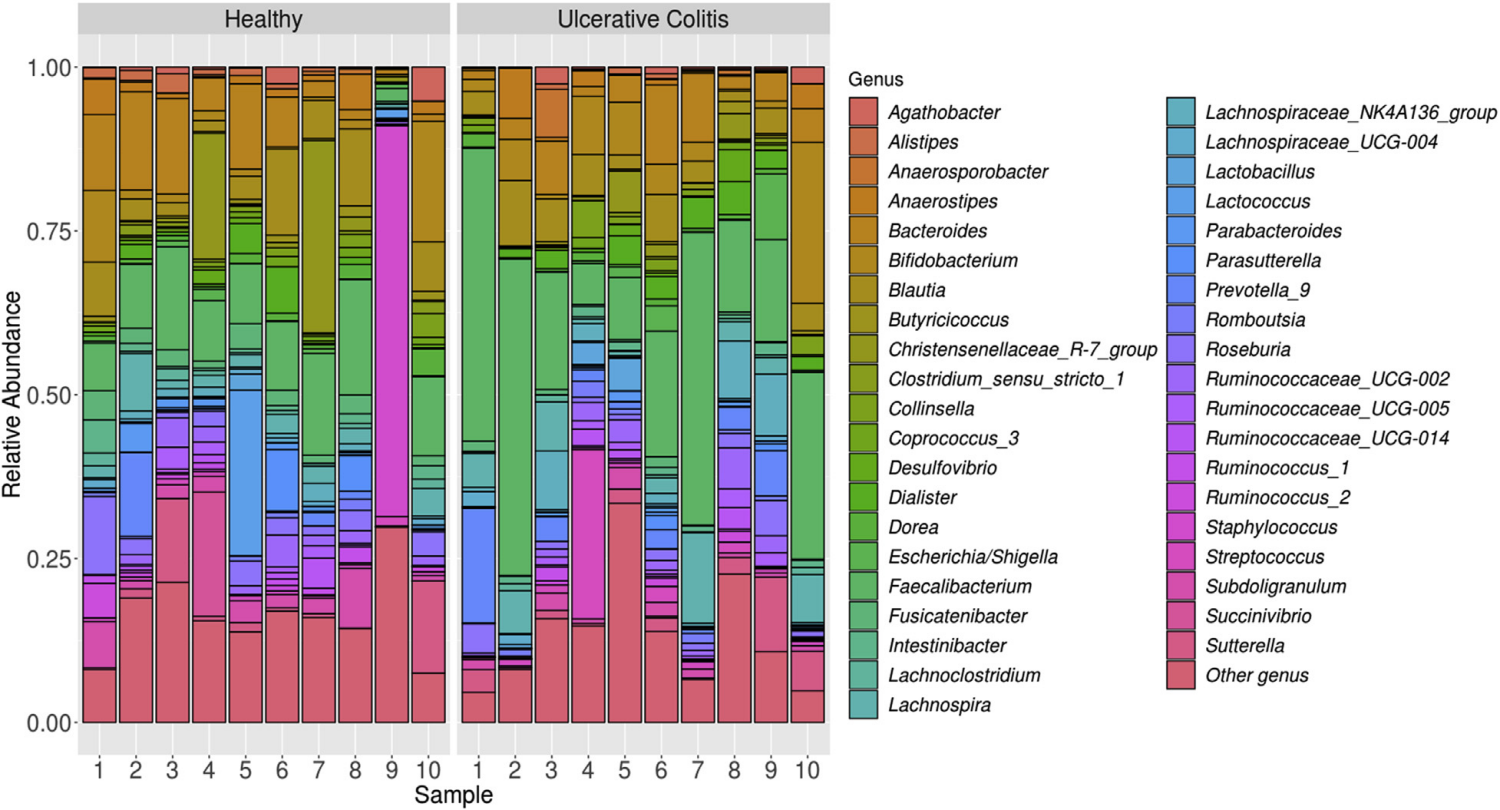
The abundance of the top 40 most common bacterial genus in each of the 20 samples.

The study involved 20 patients who were divided into 2 equal groups: 10 patients with confirmed UC and 10 from control group. We estimated the total abundance of all identified taxa based on the number of reads. Raw sequencing data is available in NCBI BioProject database (BioProject ID: PRJNA647405). Complete table of identified 40 common bacterial genera see in supplementary materials (supplementary file 1). As a result, 40 most common genera were identified (Figure 1).

**Figure 2:**
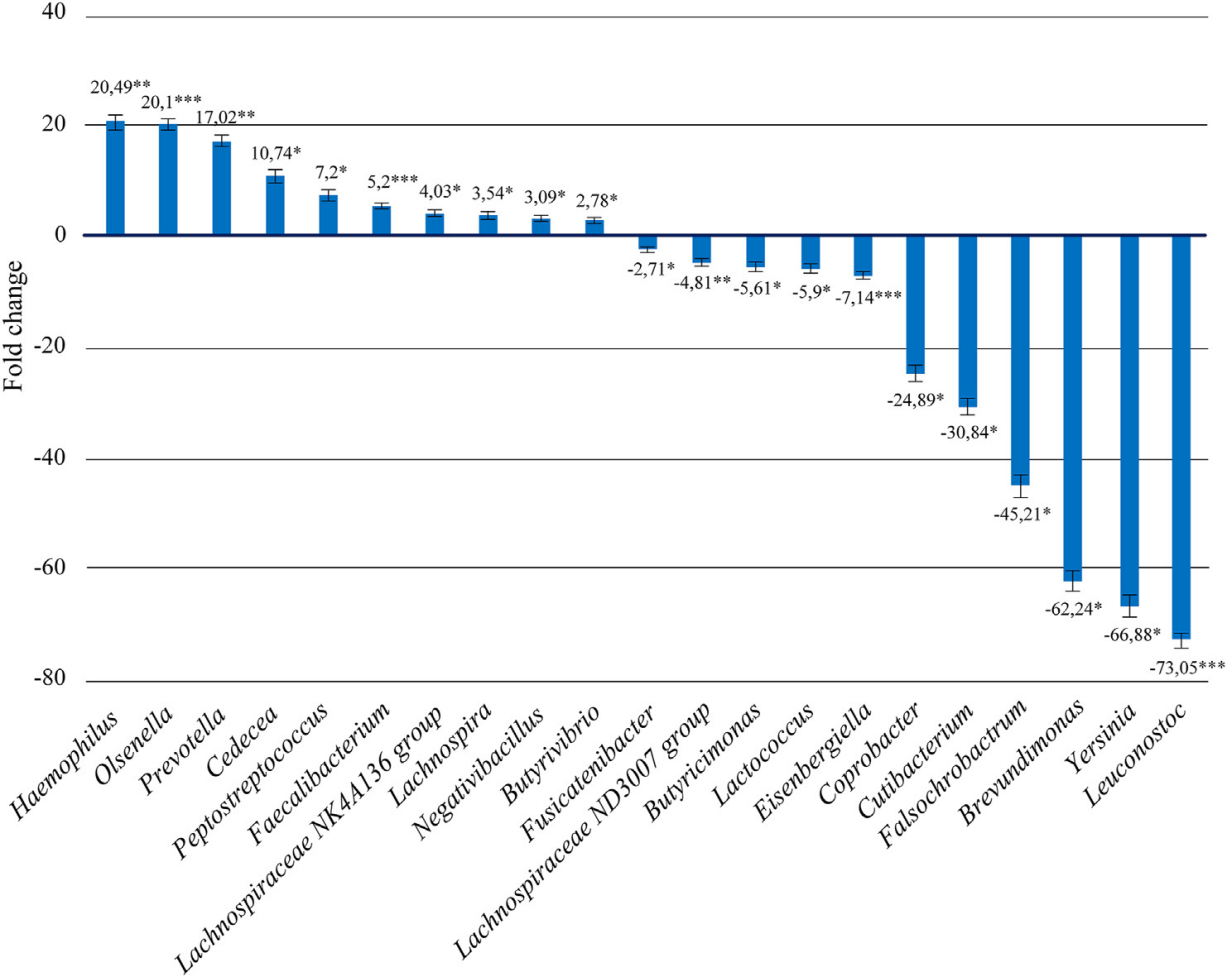
Quantitative changes in the bacteria generic composition of the intestinal microbiome in patients with ulcerative colitis relative to control group (∗Pvalue≤0.05, ∗∗Pvalue≤0.01, ∗∗∗Pvalue≤0.001).

Quantitative analysis of the generic composition of the microbiome in patients with UC showed increase in the number of bacteria from the genus *Faecalibacterium* by 5.19 times (*p* = 8.46E-05)*,* Lachnospiracea *NK4A136 group* by 4.03 times (*p* = 0.021)*, Prevotella* by 17 times (*p* = 7.47E-05)*, Lachnospira* by 3.54 times (*p* = 0.022)*, Butyrivibrio* by 2.78 times (*p* = 0.041)*, Cedecea* by 10.74 times (*p* = 0.036)*, Haemophilus* by 20.49 times (*p* = 0.01)*, Negativibacillus* by 3.08 times (*p* = 0.043)*, Olsenella* by 20.09 times (*p =* 8.46E-05)*, Peptostreptococcus* by 7.19 times (*p* = 0.036). At the same time, we observed a decrease of the genus *Fusicatenibacter* by 2.71 times (*p* = 0.047)*, Lactococcus* by 5.9 times (*p* = 0.030)*,* Lachnospiraceae *ND3007 group* by 4.80 times (*p* = 0.006)*, Eisenbergiella* by 7.13 times (*p* = 0.001)*, Leuconostoc* by 73.05 times (*p* = 0.001)*, Butyricimonas* by 5.61 times (*p* = 0.046)*, Coprobacter* by 24.88 times (*p* = 0.030)*, Yersinia* by 66.88 times (*p* = 0.030)*, Cutibacterium* by 30.83 times (*p* = 0.030)*, Brevundimonas* by 62.24 times (*p* = 0.022)*, Falsochrobactrum* by 45.20 times (*p* = 0.046)*.* All the above changes are shown in Figure 2. Complete table of identified 100 common bacterial genera see in supplementary materials (supplementary file 2).

**Figure 3:**
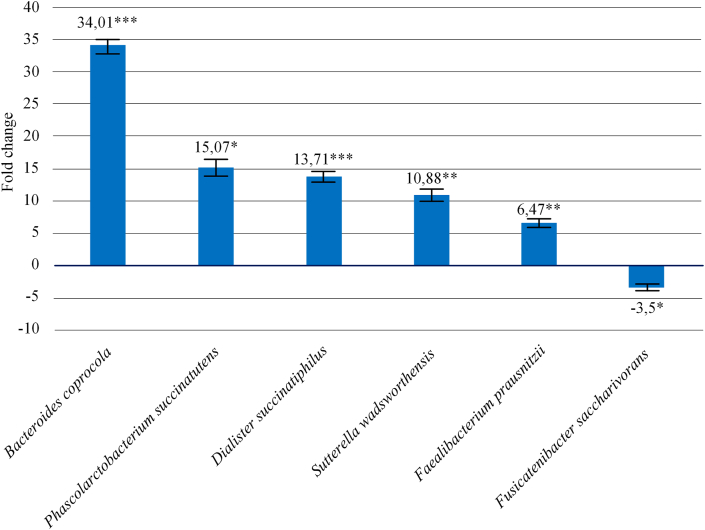
Changes in species composition of intestinal microbiome in patients with ulcerative colitis relative to control group (∗Pvalue≤0.05, ∗∗Pvalue≤0.01, ∗∗∗Pvalue≤0.001).

Moreover, analysis of fecal microbiota revealed that patients with UC showed a significant increase in the level of species *Faecalibacterium prausnitzii* by 6.47 times (*p* = 0.005), *Sutterella wadsworthensis* by 10.88 times (*p* = 0.008), *Dialister succinatiphilus* by 13.71 times (*p* = 0.001), *Bacteroides coprocola* by 34.0 times (*p* = 0.001) and *Phascolarctobacterium succinatutens* by 15.07 times (*p* = 0.033) in biological samples compared to the control group, and a decrease in the bacteria *Fusicatenibacter saccharivorans* by 3.50 times (*p* = 0.014). All the above changes are shown in Figure 3.

## 4 Discussion

In this work, we studied the microbiological composition of the intestines of patients with UC in the Central European part of Russia and compared the data with the control group. It was shown significant differences between study groups. Thus, our study showed that the number of bacteria of the genus *Haemophilus* was increased in patients with UC. *Haemophilus* is a genus that consists of Gram-negative bacteria, mainly commensal organisms, although there are pathogenic species, such as *H. influenzae* or *H. ducreyi*. According to the literature, several types of bacteria belonging to the genus *Haemophilus*, such as *H. parainfluenzae*, may be associated with the development of inflammatory diseases, including UC [25].

The genus *Olsenella* most often colonizes the human oral cavity and leads to the development of endodontic infection, however, in 2019, anaerobic bacteria of the genus *Olsenella* were isolated from the feces of healthy people [26]. We observed an increase in bacteria of the genus *Olsenella* in patients with UC. This may be due to the fact that the majority of members of this genus produce lactic acid as the final metabolit, which increase production has pathogenetic significance in UC [27].

The genus *Prevotella* includes Gram-negative bacteria, which are classically considered commensal bacteria due to the extensive presence in the healthy human body and the rare involvement in infections. However, new studies have linked an increase in the number of these bacteria with inflammatory disorders, suggesting that some strains have pathogenetic properties. According to some studies, an increase in *Prevotella* can cause inflammatory diseases, including UC [28]. Our study confirms these data, since we observed an increase in the number of this genus in UC. However, there is a need for more studies in humans to ascertain a casual and potential disease-triggering role for *Prevotella*. Inflammatory diseases are highly heterogeneous and develop through the complex interaction between host genetic risk factors and environmental exposures. Thus *Prevotella* may only play a part in certain disease endotypes, and larger cohort studies are needed to delineate causal relationships [28].

*Cedecea* is a genus of Gram-negative bacteria is extremely rare, comprising only 5 species. According to our data, the number of these bacteria increases in patients with UC. These data may indicate the possible pathogenicity of some types of bacteria of this genus, but their role in the disease is not yet known.

We also revealed an increase of the number of the genus *Peptostreptococcus* in patients with UC compared with normal values. The result is consistent with other experimental data, according to which an increase in bacteria of the genus *Peptostreptococcus*, which are potential pathogens, leads to dysbiotic disorders in the human intestine. A dysbiotic microbiota leads to the loss of normal regulatory immune effects in the intestinal mucosa, and further to the development and maintenance of the inflammatory process [29].

Bacteria of the genus *Faecalibacterium* are one of the most common anaerobic bacteria of the human intestinal microbiota, which accounts for about 5% of the total number of bacteria in feces. They play an important role in providing energy to colonocytes and maintaining intestinal health [30]. According to available data, a lack of these bacteria can cause and increase inflammation. In particular, a significant inverse correlation between disease activity and the amount of *Faecalibacterium* is detected in patients with UC [31]. However, our data showed an increase of this genus in patients with UC. These data may be associated with population characteristics of the microbiome composition, but this hypothesis will be studied [32].

Quantitative analysis revealed a decrease of the genus *Butyricimonas* in patients with UC. Literature data describe a decrease in the abundance of genus *Butyricimonas* in inflamed areas of the intestinal mucosa in patients with UC compared to patients without IBD [33]. Thus, the obtained data show that the genus *Butyricimonas* can play an important role in the pathogenesis of UC.

In our study it was found that, the number of bacteria of the genus *Lactococcus* decreases in UC. It is known that representatives of the genus *Lactobacillus* have therapeutic properties such as improvement of normal microbiota, prevention of infectious diseases and food allergies, modulation of innate and adaptive immune response [34].

According to our research results, the number of *Eisenbergiella* bacteria decreases in patients with UC. According to the literature, *Eisenbergiella massiliensis* was isolated from stool samples of a French woman suffering from obesity, but no quantitative analysis of the microbiome composition in the sample was conducted [35]. There is also evidence that representatives of this genus are abundant in the intestinal microbiome of professional bodybuilders, which may be due to their high-protein diet [36]. Thus, our study shows for the first time an increase of *Eisenbergiella* sp*.* bacteria in patients with UC.

The number of the genus *Coprobacter* bacteria decreases in UC. It is known that representatives of the genus were previously isolated from the feces of healthy people [37], but there is no data describing their role in the development of any intestinal pathology.

Also, the number of representatives of the genus *Cutibacterium* was reduced in patients with UC, relative to normal value. No literature data were found on the relationship of the genus *Cutibacterium* to the development of IBD, including UC. However, it is known that this genus, all representatives of the *Propionibacteriaceae* family, is a producer of propionic acid. According to literature data, propionic acid has antitumoral and anti-inflammatory effects, its decrease was noted in feces of patients with IBD, including UC [38]. Thus, it can be assumed that the decrease of propionovoxid bacteria in the human intestinal microbiome may be one of the factors of UC development, which reflects the data of our study.

Representatives of the genus *Brevundimonas* are gram-negative aerobic bacilli, which are widely distributed in the environment, but rarely isolated from clinical samples [39]. During sequencing and subsequent data analysis, we identified the *Brevundimonas* genus, which were reduced in UC patients. There is evidence of hospital-acquired infection with *Brevundimonas* bacteria in immunocompromised humans, but no patients with intestinal disease have been reported [40]. Thus, the role of the *Brevundimonas* genus in the intestinal microbiome interaction system is unclear.

*Yersinia* is a genus of Gram-negative bacteria, which in small amounts can be part of the human gut microbiome [41]. This genus includes 18 species, some of which are pathogenic to humans and can lead to the development of infectious diseases. However, despite the fact that the infectious factor plays a significant role in the development of the IBD, many studies have disproved the direct role of infectious agents of the *Yersinia* genus in the development of UC [42]. Our experiment showed a decrease in the number of *Yersinia* genus representatives in UC patients, which is consistent with the available literature data.

The *Leuconostoc* genus is represented by gram-positive, facultatively anaerobic, non-transmitting bacteria producing lactic and acetic acid [27]. However, according to the literature, there is no consensus on the role of this species in the development of UC. Some data demonstrate an increase in the number of *Leuconostoc*, while others, on the contrary, show a decrease in its number in UC [43]. Our study showed that in patients with UC the number of bacteria of the genus *Leuconostoc* was reduced. Taking into account the fact that there is no consensus on the contribution of the genus *Leuconostoc* to the development of UC, further research is needed to determine their impact on human health.

The number of *Faecalibacterium prausnitzii* bacteria in patients with UC was higher than in healthy individuals. According to the literature data *F. prausnitzii* is one of the main butyrates-producing bacteria from clostridial cluster IV, having anti-inflammatory and immunostimulating effect [7]. Its number decreases in IBD, including in UC, as well as in colorectal cancer, celiac disease, and irritable bowel syndrome. According to some data this may be due to an increase in the ratio of *Bacteroides fragilis* to *Faecalibacterium prausnitzii* [43]. In our data, there was no decrease of *F. prausnitzii* bacteria in patients with UC in comparison with healthy group. We suppose that this may be due to population peculiarities of microbiome composition. Therefore, there is a need to continue studying the quantitative changes of microbiome composition, in particular the bacteria of the species *F. prausnitzii* in UC, as well as other types of IBD.

The quantitative indicator of *Fusicatenibacter saccharivorans* in patients with UC was lower than normal, which is consistent with the literature data, which also shows the anti-inflammatory effect of *F. saccharivorans* [44].

The content of *Dialister succinatiphilus* in biological samples of patients with UC exceeds normal parameters. According to literature data, *D. succinatiphilus* bacteria are capable of producing succinate and acetic acid [45]. The increase in microbial production of these metabolites is of pathogenetic importance in UC, as they act as pro-inflammatory signal molecules [27].

In our study, the largest deviation from the norm was shown for the species *Bacteroides coprocola*. We were not able to find data describing the relationship of this species with the development of UC or other IBD. However, analysis of the 16S rRNA sequence of *B. coprocola* showed the identity of this strain by 92.7% with the species *Bacteroides vulgatus* in one of the studies. *B. vulgatus* most frequently and in larger quantities has been found in the colon tissue samples obtained from patients with UC [46]. According to the authors of the study, although *B. vulgatus* is not a direct cause of IBD, it can significantly affect the course of the disease, for example, by slowing remission [43].

Also, the bacterial species *Sutterella wadsworthensis* and *Phascolarctobacterium succinatutens* exceeded the normal values in the samples of patients with UC, respectively. According to the literature, the species *S. wadsworthensis* was previously found in intestinal microbiota analysis in patients with UC, but experimental data did not confirm a connection between *S. wadsworthensis* and the pathogenesis of this disease and other types of IBD [47].

The literature data we studied the role of *Phascolarctobacterium succinatutens* did not cover in the development of any types of IBD, including UC. *P. succinatutens* is known to be able to utilize succinate and produce propionate [48], which has anti-inflammatory and antitumor effects [49]. In this regard, further research is needed to determine the contribution of *P. succinatutens* to the system of metabolic interaction of intestinal microbiota.

For high-throughput sequencing, we selected the V5–V6 region of 16S rRNA. It was shown early that V4–V6 sub-region was the most reliable for the full-length 16S rRNA sequences in the phylogenetic analysis of most bacterial phyla [50]. Chakravorty et al. analyzed V3 and V6 region sequences from 110 bacteria that infect humans and showed that the V6 region is the best choice for distinguishing between bacterial species [51]. In another study it was shown that OTU richness is much higher with the V6 tag than with the V4 tag [52].

## 5 Conclusion

In this research we assessed microbial changes associated with UC in a group of treatment-naive patients. Healthy patients without UC were used for a control group. Statistically significant changes were detected at the genus and species taxa in patients with UC compared with the control group. In our study we observed a significant increase of genus: *Haemophilus, Olsenella, Prevotella, Cedecea, Peptostreptococcus, Faecalibacterium, Lachnospira, Negativibacillus, Butyrivibrio*, and species: *Bacteroides coprocola, Phascolarctobacterium succinatutens, Dialister succinatiphilus, Sutterella wadsworthensis, Faecalibacterium prausnitzii*. At the same time, there was a significant decrease in the genus of *Fusicatenibacter, Butyricimonas, Lactococcus, Eisenbergiella, Coprobacter, Cutibacterium, Falsochrobactrum, Brevundimonas, Yersinia, Leuconostoc* and in the species of *Fusicatenibacter saccharivorans*. Actually, we found confirmation of our data for genus *Haemophilus, Olsenella, Prevotella, Peptostreptococcus, Butyricimonas, Lactococcus, Fusicatenibacter saccharivorans* and *Dialister succinatiphilus* in other literary sources and studies of UC. For some taxa we did not find data confirming their relationship with the development of UC or other IBD or these data were ambiguous. Moreover, we discovered a few taxa such as *Negativibacillus* and *Falsochrobactrum* that have not previously been found in human stool samples. We received unexpected data for *Faecalibacterium* and *Faecalibacterium prausnitzii*. Their number decreases with UC according to many published data, but we got opposite results. While we do not fully understand the nature of these discrepancies, perhaps this is due to the population characteristics of the studied sample. Our evidence confirm that more research is needed to understand the role of microbiome changes in the development IBD in particular UC. At the moment, we cannot make clinical recommendations for the prevention and treatment of UC based on the data obtained, since a more detailed study of population characteristics and microbiome characteristics is needed. We expect that future studies will provide a comprehensive view of how the microbiome and its complex constituents interact with the host to influence health and development of disease. In the future, we plan to investigate the effect of probiotics and prebiotics and their combinations on the microbiological composition of the intestines of UC patients.

In general, our data suggest that more attention should be paid to the study of the gut microbiota. It is necessary to take into account the nationality of the patient, since the microbiota pattern may differ in patients with ulcerative colitis in relation to healthy people of the same nationality. In addition, the data obtained by us show which taxonomic groups of bacteria may be responsible for the appearance of ulcerative colitis.

## Declarations

### Author contribution statement

M.V. Gryaznova, Yu.D. Dvoretskaya, A.V. Panevina: Performed the experiments; Wrote the paper.

S.A. Solodskikh: Conceived and designed the experiments; Performed the experiments.

M.Y. Syromyatnikov: Conceived and designed the experiments; Analyzed and interpreted the data; Wrote the paper.

T.N.Sviridova: Analyzed and interpreted the data; Contributed reagents, materials, analysis tools or data.

E.S. Popov: Conceived and designed the experiments; Analyzed and interpreted the data.

V.N. Popov: Conceived and designed the experiments; Analyzed and interpreted the data; Contributed reagents, materials, analysis tools or data.

### Funding statement

This work was supported by 10.13039/501100006769Russian Science Foundation (project 19-76-10023) and 10.13039/501100012190Ministry of Science and Higher Education of the Russian Federation in the framework of the national project “Science” (project FZGW-2020-0001, unique number of the register of State tasks 075001X39782002).

### Data availability statement

Data included in article/supp. material/referenced in article.

### Competing interest statement

The authors declare no conflict of interest.

### Additional information

No additional information is available for this paper.

## Appendix A Supplementary data

Supplementary_file_1.xlsx Supplementary_file_1.xlsx

Supplementary file 2.xlsx Supplementary file 2.xlsx
